# Multidimensional evaluation of tracheobronchial disease in adults

**DOI:** 10.1007/s13244-016-0489-2

**Published:** 2016-04-16

**Authors:** Susan E. G. Sims, Faqian Li, Thomas Lostracco, Abhishek Chaturvedi, Hongju Son, John Wandtke, Susan Hobbs

**Affiliations:** Department of Imaging Sciences, University of Rochester Medical Center, 601 Elmwood Avenue, Box 648, Rochester, NY 14642 USA; Department of Laboratory Medicine and Pathology, University of Minnesota, MMC 76, C420 Mayor Building, 420 Delaware Street, NE, Minneapolis, MN 55455 USA; Department of Radiology, Einstein Healthcare Network, 5501 Old York Road, Philadelphia, PA 19141 USA

**Keywords:** Computed tomography, Pathology, Trachea, Bronchi, Adult

## Abstract

The large airways can be affected by a wide spectrum of acquired benign and malignant diseases. These lesions may present as focal or diffuse processes and with narrowing or widening of the airway. Some of these may be asymptomatic for quite some time and may be incidentally detected on imaging, while others may be symptomatic, causing airway compromise. There may be a characteristic radiograph and computed tomography (CT) appearance, suggesting a narrow differential. When the imaging findings are not definitive, tissue may be obtained for pathological analysis. It behooves the radiologist to be familiar with the pathologic findings that correlate with the radiographic or CT appearance of the most frequently seen large airway lesions. In this way, we may improve our diagnostic accuracy. This paper will present the imaging findings of the most prevalent tracheobronchial lesions along with any associated pathology.

*Teaching Points*

• *The large airways can be affected by many acquired benign and malignant diseases*.

• *Large airway lesions may present as focal or diffuse processes, with narrowing or widening*.

• *There may or may not be characteristic imaging appearance of large airway disease*.

• *If imaging findings are not definitive, tissue may be obtained for pathological analysis*.

## Introduction

There is a wide range of lesions found on imaging of the large airways. These lesions can fall into a number of categories, namely benign or malignant, focal or diffuse, and narrowing or widening of the airway. Observation of the morphology of the lesion on radiographs and computed tomography (CT) can often narrow the differential substantially; therefore, knowledge of the radiographic presentation of various large airway masses can be instrumental in determining the next step in management.

**Fig. 1:**
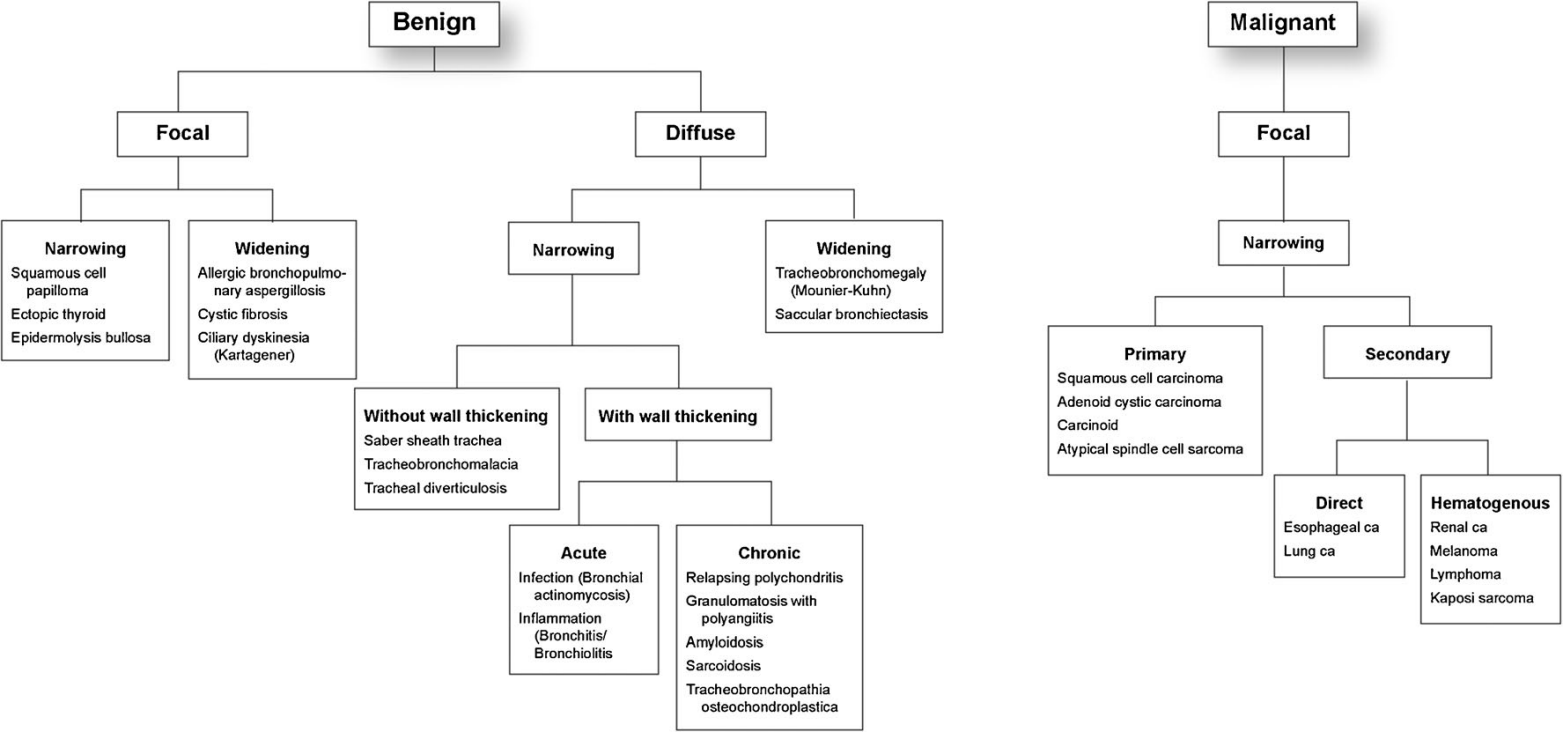
Benign tracheobronchial lesions organized by radiological morphology: focal vs. diffuse, narrowing vs. widening, acute vs. chronic. Malignant tracheobronchial lesions organized by radiological morphology: focal (no relevant lesions in the diffuse category), narrowing (no relevant lesions in the widening category), primary vs. secondary, direct vs. hematogenous

The imaging characteristics of various tracheobronchial masses are often nearly definitive in determining the diagnosis; however, if not, then tissue may be obtained for pathological analysis. It behooves the radiologist to be familiar with the pathologic findings that correlate with the radiographic or CT appearance of the most frequently seen large airway lesions. In this way, we may improve our diagnostic accuracy. This paper will present the imaging findings of the most prevalent tracheobronchial lesions along with the associated pathology. We have chosen to organize these lesions first as benign versus malignant, then by radiological morphology such as focal/diffuse and narrowing/widening of the airway (Fig. 1).

## Anatomy

The trachea conveys air from the larynx to the bronchi. In addition, it assists with humidification and warming of the inspired air and with mucocilliary clearance [1]. It extends from the level of the C6 vertebra to its bifurcation at the level of T5 and is approximately 11 cm in length [2]. There are 15–20 C-shaped hyaline cartilage segments that maintain lumen patency.

The tracheal wall constitutes of the inner mucosa, sub-mucosa, cartilage, longitudinal and transverse muscle fibres, and an outer adventitial layer. The endoluminal diameter in the coronal plane ranges from 13 to 25 mm in men and 10 to 21 mm in women. The sagittal diameter ranges from 13 to 27 mm in men and 10 to 23 mm in women [3].

At the tracheal bifurcation, the right mainstem bronchus emerges as a shorter, more vertically oriented structure with a larger diameter than the left mainstem bronchus. The right and left mainstem bronchi form 20–30° and 45° angles with the trachea, and are approximately 2.5 and 5 cm long, respectively [4].

## Lesions of the large airways

### Benign – focal – narrowing

#### Squamous cell papilloma

**Fig. 2:**
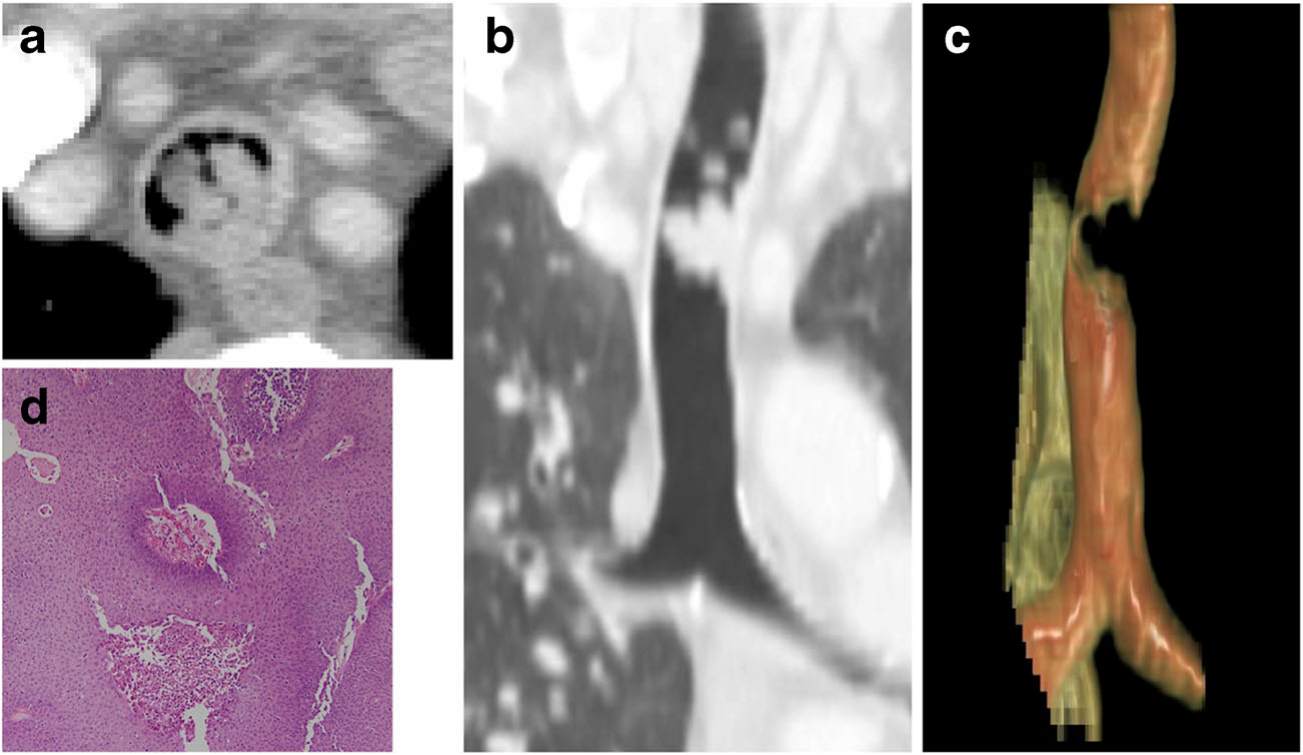
Seventy-seven-year-old woman with squamous cell papilloma of the trachea. **a** Axial and **b** coronal CT imaging demonstrating frond-like polypoid masses extending into the tracheal lumen. **c** 3D VR image shows the large extent of the lesion and the significant airway narrowing. **d** Pathology reveals papillary squamous cell proliferation with fibrovascular core and bland morphology

Benign tumours of the trachea are exceedingly rare. One of the most common of these rare tumours is squamous cell papilloma. On CT, there may be multiple protrusions into the airway or nodular thickening of the airway leading to intrinsic narrowing (Fig. 2). Tracheobronchial papillomatosis describes the presence of multiple squamous cell papillomas involving the trachea and bronchi. These tumours are usually well defined with smooth surfaces. In addition to the findings within the airways, CT examination may depict cysts with adjacent nodules within the pulmonary parenchyma, findings associated with distal spread [5]. Squamous cell papilloma has a bimodal age distribution: in children it is commonly acquired secondary to contact with the human papilloma virus (HPV) during transit through the birth canal and in adults through orogenital contact [6]. Squamous cell papilloma can undergo malignant transformation, which has been reported in 10 % of adult cases [6]. This transformation increases with age, smoking, and certain serotypes of HPV.

#### Ectopic thyroid

**Fig. 3:**
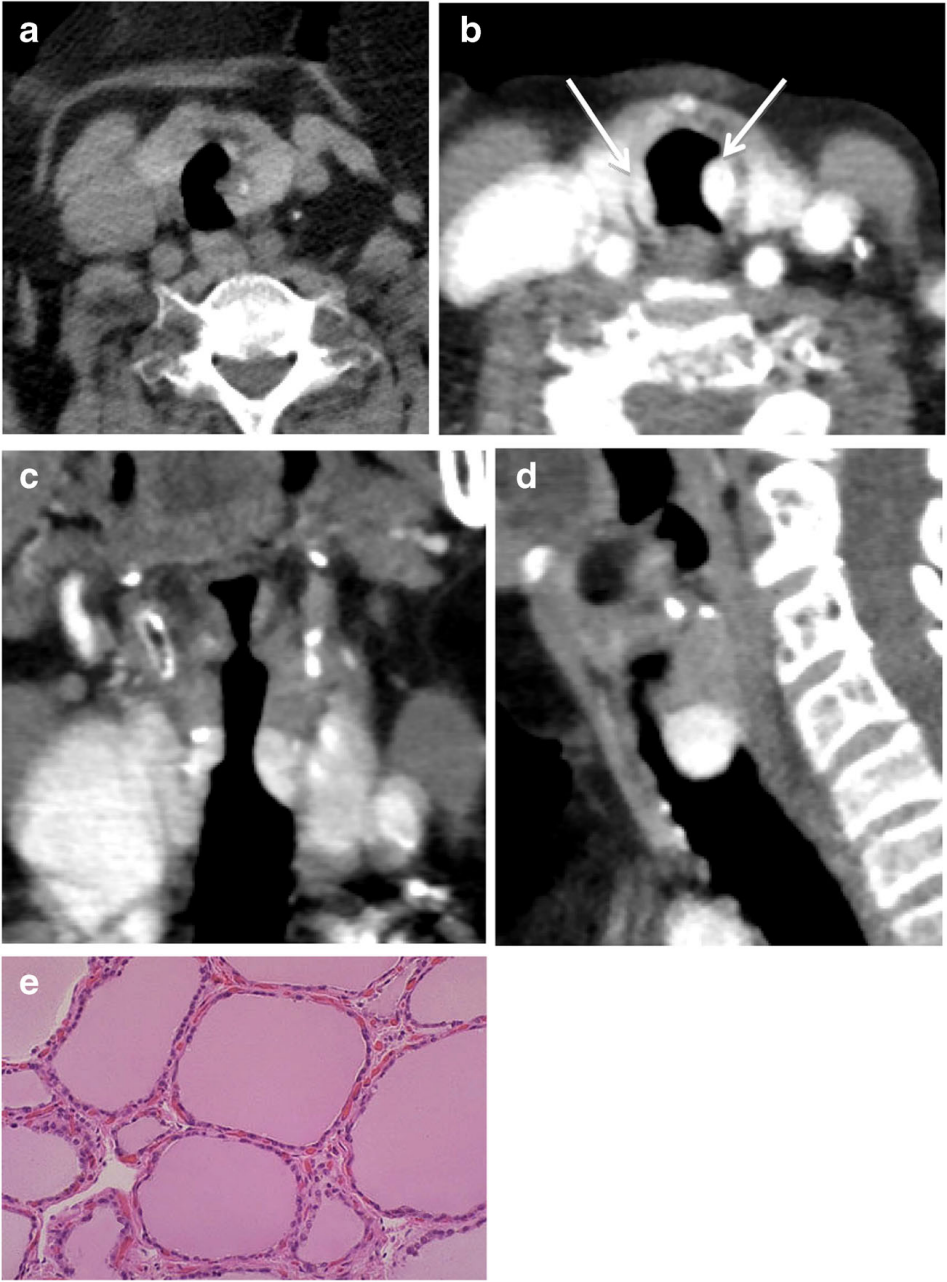
Seventy-nine-year-old woman who presented with stridor. **a** Axial CT image acquired without intravenous contrast followed by **b** axial, **c** coronal, and **d** sagittal intravenous contrast enhanced imaging. There is avidly enhancing endoluminal tissue that enhances similarly to the thyroid gland at the same level (**b**, *arrows*). **e** Pathology of the enhancing soft tissue within the trachea was ectopic thyroid tissue with thyroid follicles of different size

Ectopic thyroid tissue is usually found along the course of the thyroglossal duct [7], and may be seen rarely in locations outside this region, such as within the trachea (Fig. 3). Typically, the patient presents with stridor, which may be incorrectly diagnosed as asthma [8]. On CT the attenuation of such a lesion will be similar to that of normal thyroid on unenhanced and enhanced CT. Up to 11 % of ectopic thyroid lesions will undergo malignant transformation, most commonly to papillary thyroid carcinoma [9]. Biopsy is required for definitive diagnosis.

#### Epidermolysis bullosa

**Fig. 4:**
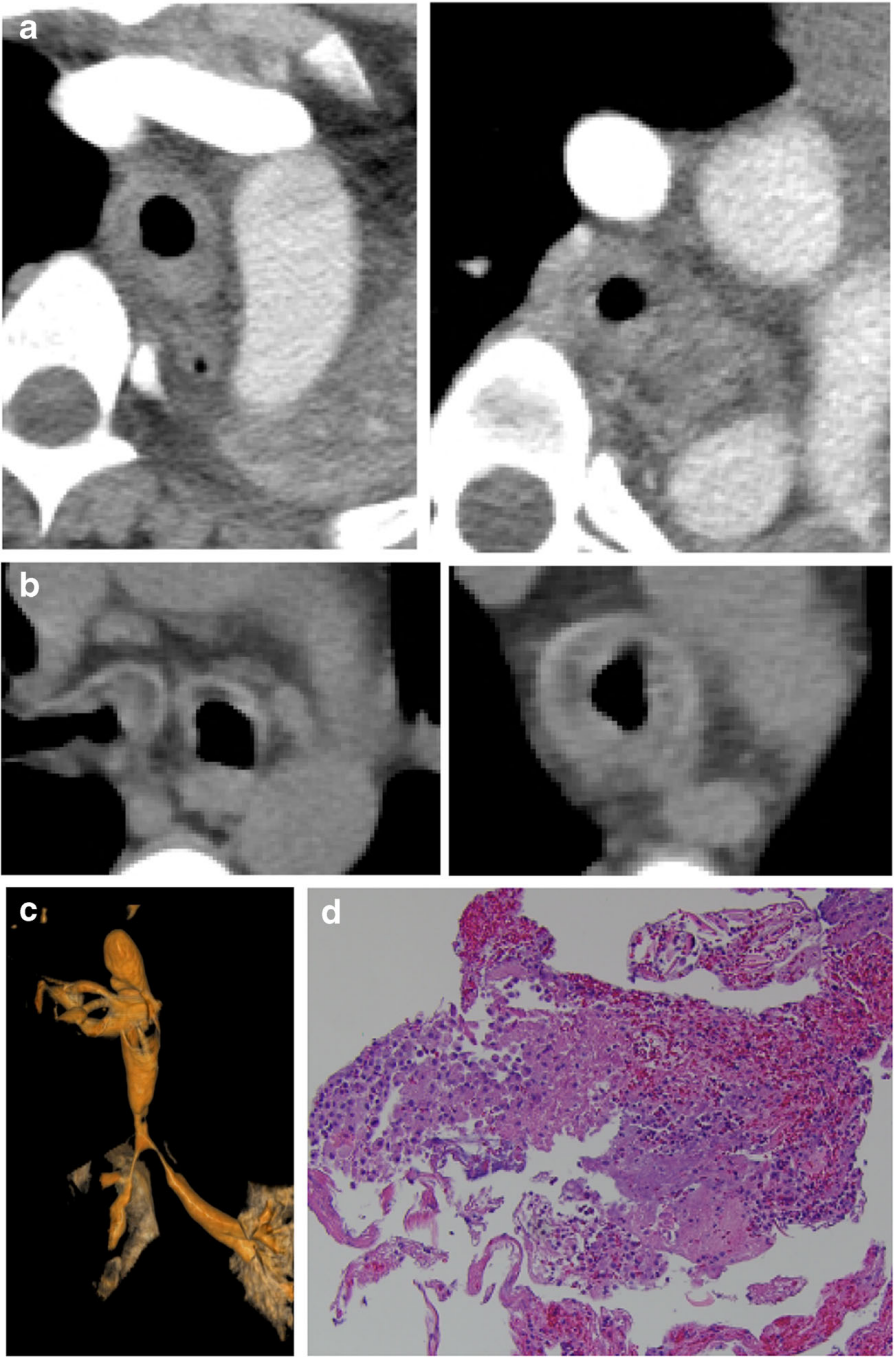
Twenty-five-year-old woman with airway stricture. **a** Contrast-enhanced axial CT and **b** non-contrast axial CT obtained 1 year later demonstrating non-enhancing focal narrowing of the distal trachea and proximal main stem bronchi. **c** 3D VR image. **d** Pathology reveals inflammatory exudate with fibrin and neutrophils compatible with epidermolysis bullosa

Epidermolysis bullosa is an inherited connective tissue disease that manifests as blistering of the skin and/or the mucosa following minor trauma. It most commonly affects the oesophageal mucosa, but can also be found in the trachea, presenting as diffuse circumferential thickening with narrowing of the trachea (Fig. 4).

#### Iatrogenic

**Fig. 5:**
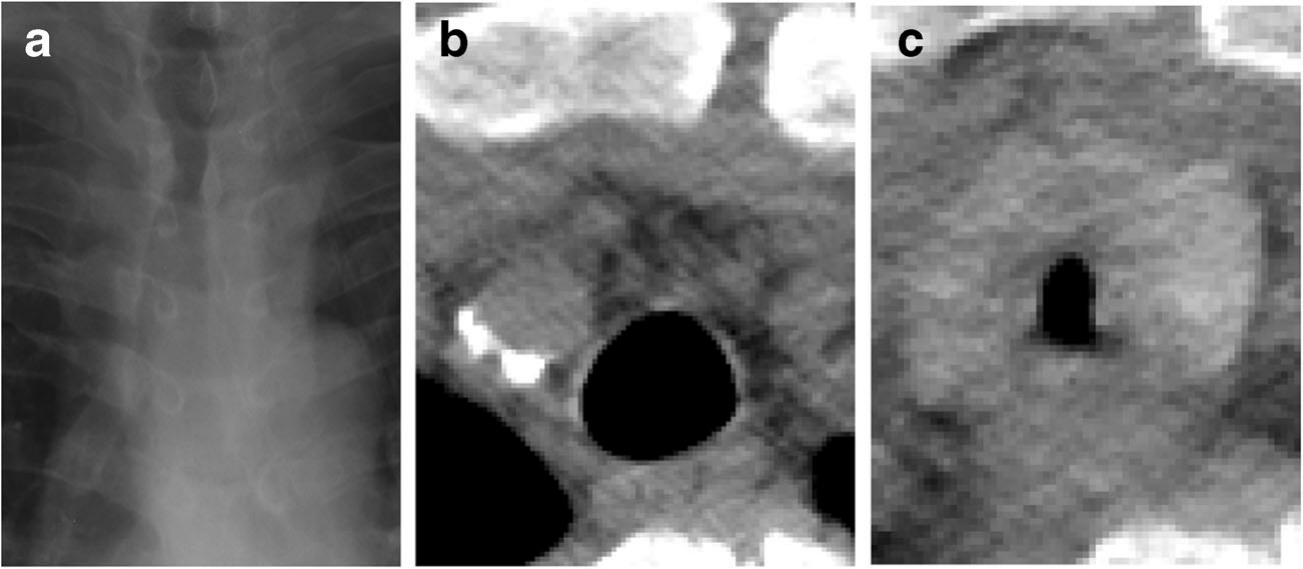
Fifty-two-year-old man with history of traumatic brain injury and extended intubation. **a** Chest radiograph for a syncopal episode demonstrating an hourglass shape of the thoracic trachea. **b** Pre- and **c** Post-intubation axial CTs of the same patient demonstrating interval focal narrowing of the trachea, likely iatrogenic

Post intubation stenosis is an example of focal tracheal narrowing. A similar injury can occur with the placement of a tracheostomy tube. The high pressure of the endotracheal tube balloon can lead to mucosal necrosis resulting in scarring and stenosis [10]. The advent of low pressure cuffs has reduced the prevalence of stenosis to less than 1 % [6]. Endotracheal tube-related stenosis occurs in the subglottic region. Tracheostomy-related narrowing occurs at the stoma site. The typical appearance on plain film is an hourglass shape demonstrating narrowing of the thoracic trachea [11] (Fig. 5).

#### Substernal goiter

**Fig. 6:**
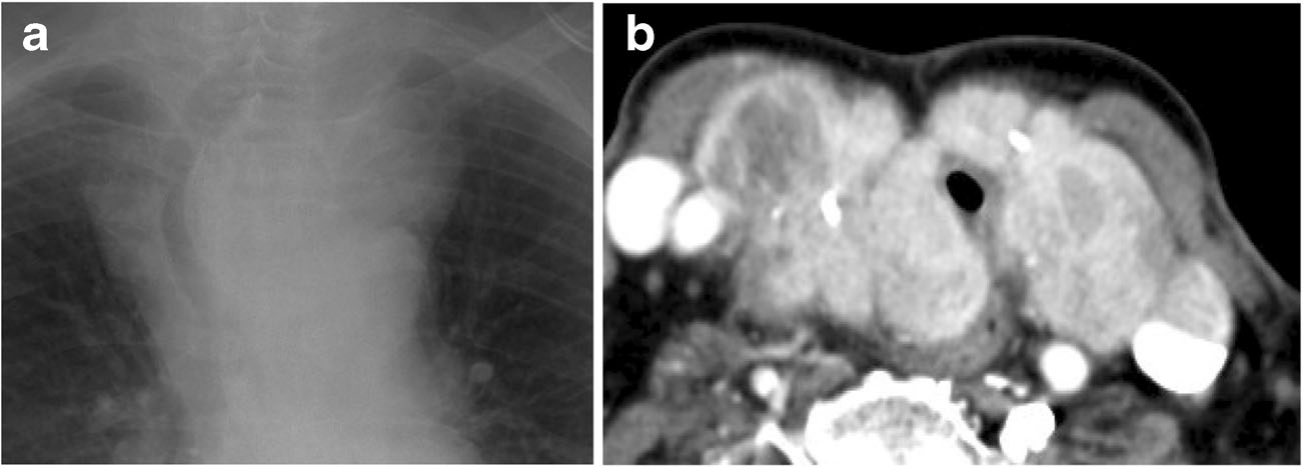
**a** Radiograph demonstrates tracheal deviation and narrowing at the level of the thoracic inlet. **b** Axial CT image demonstrates a heterogenous, high attenuating mass with calcifications, consistent with a goiter

Substernal goiter is a one of the most common causes of external compression of the trachea [12]. Clinical symptoms of external compression of the trachea by a substernal goiter include dyspnoea, wheezing, and cough. It can present great difficulty during intubation. If obstructive symptoms become severe, surgical resection may be performed. Imaging findings can include tracheal deviation and narrowing of the thoracic inlet (Fig. 6).

### Benign – focal – widening

#### Allergic bronchopulmonary aspergillosis

**Fig. 7:**
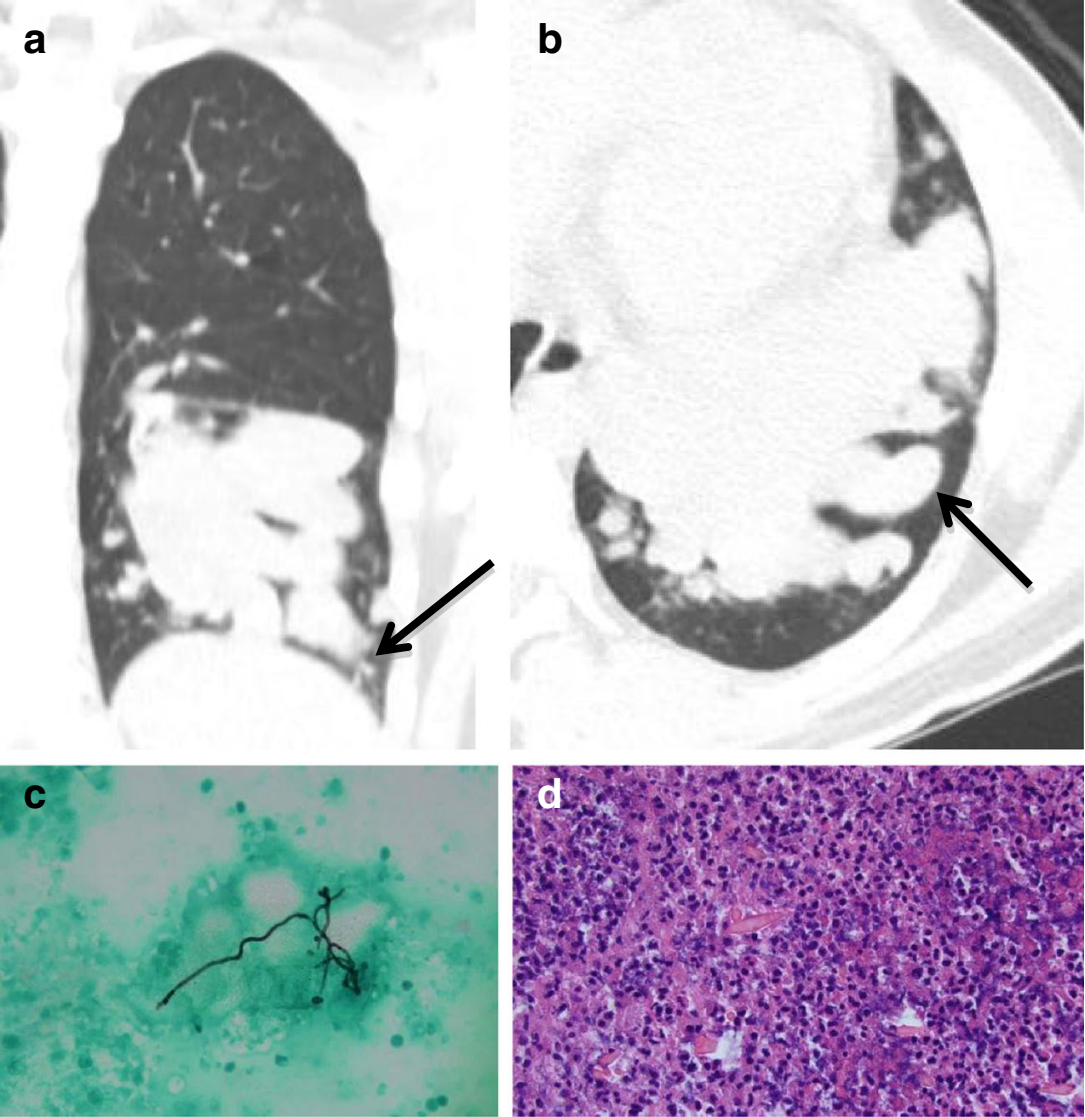
Twenty-two-year-old man. **a** Coronal and **b** axial CTs demonstrating numerous dilated bronchi within the left lower lobe, filled with mucous secretions (“finger-in-glove”, *arrows*), consistent with bronchiectasis and superimposed infection. Pathology reveals **c** fungal hyphae morphologically consistent with *Aspergilla* species and **d** acute inflammatory infiltrates and necrotic debris

Allergic bronchopulmonary aspergillosis usually presents in patients with a pre-existing pulmonary disease such as asthma [13]. It is a result of hypersensitivity to aspergillus and is considered an eosinophilic lung disease. Treatment includes asthma management, anti-fungal medication, and corticosteroids. The CT appearance may demonstrate numerous dilated bronchi filled with mucous secretions (“finger-in-glove”) (Fig. 7) [14].

### Benign – diffuse – narrowing – without wall thickening

#### Saber sheath trachea

**Fig. 8:**
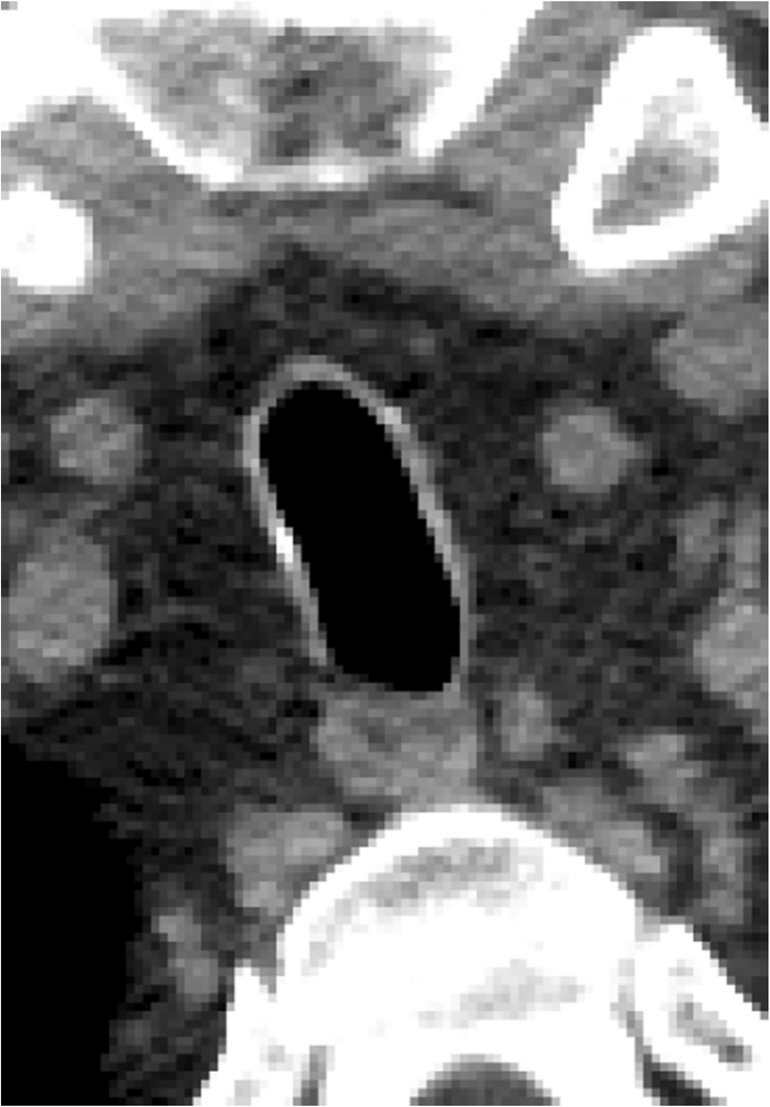
Sixty-two-year-old man with history of COPD. Axial CT in soft tissue window demonstrating intrathoracic saber sheath trachea, with a reduction in the coronal diameter of the trachea relative to the sagittal diameter

Saber sheath trachea is seen within the intrathoracic trachea, demonstrating a reduction in the coronal diameter and thickening of the trachea relative to the sagittal diameter [15] (Fig. 8). It is commonly defined as a fixed deformity of the trachea in which the coronal:sagittal diameter ratio is less than 0.5 measured at 1 cm above the aortic arch. Expiratory images will often demonstrate a further decrease in coronal diameter [11]. The patient almost always suffers from emphysema/COPD [5, 12], which is thought to contribute to saber sheath trachea through increased intrapleural pressure and tracheal injury secondary to chronic cough [3].

#### Tracheobronchomalacia

**Fig. 9:**
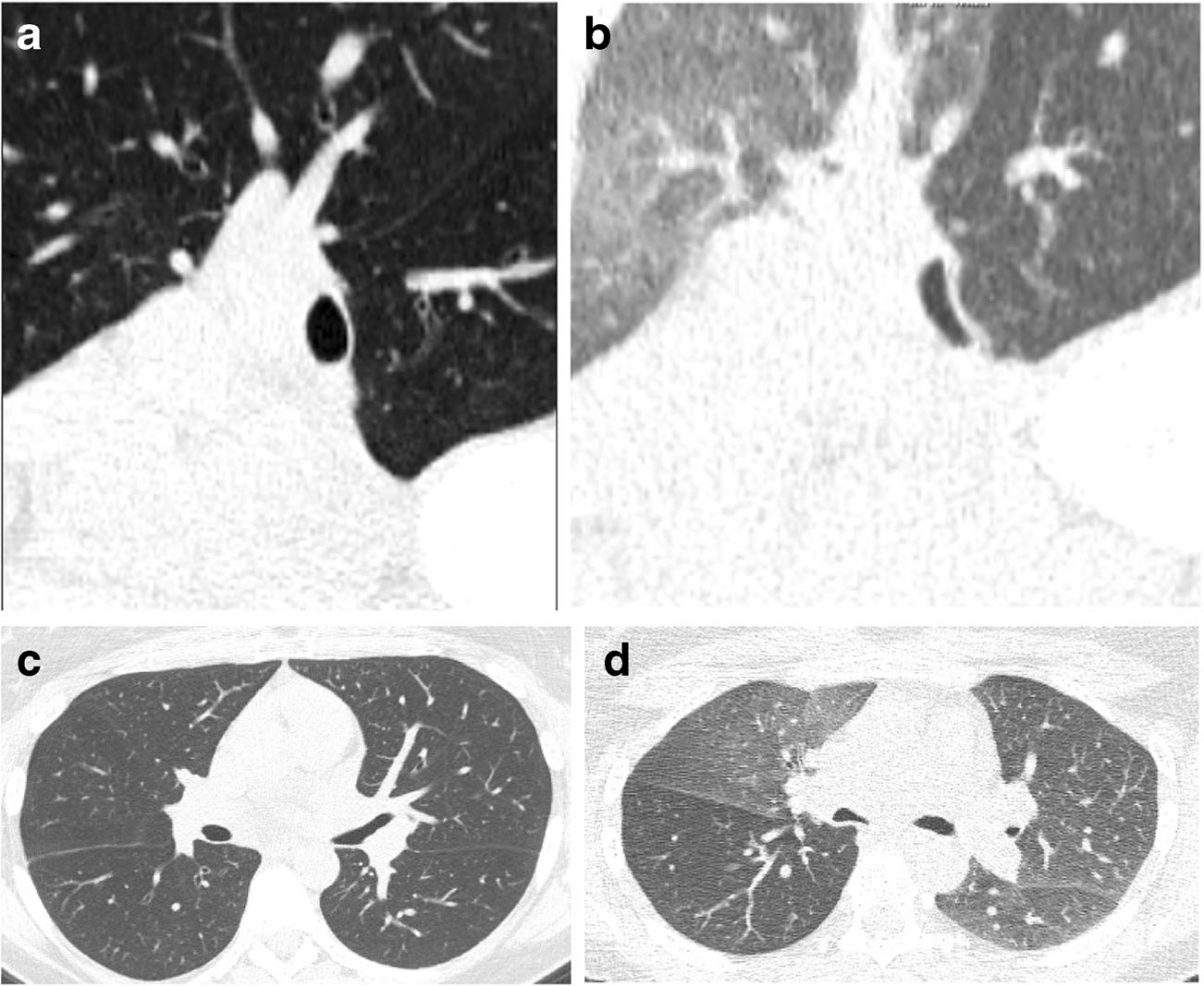
Thirty-four-year-old woman with history of atypical interstitial pneumonia status post bronchoscopy presenting with tracheobronchomalacia of the right bronchus intermedius. **a** Double oblique inspiratory and **b** expiratory CT imaging demonstrating reduced bronchial diameter on expiration. **c** Axial inspiratory CT imaging demonstrating air trapping on expiration **d**

Tracheobronchomalacia is defined as collapse (greater than 50–70 %) of the tracheobronchial wall with expiration [12] (Fig. 9). This collapse is secondary to increased intrathoracic pressure on a weak tracheal wall. The most common cause is intubation injury; less common causes include congenital lesions, extrinsic compression, chronic infection, COPD, saber sheath trachea, relapsing polychondritis, and tracheobronchomegaly [16]. Treatment options include stenting, tracheoplasty, tracheal replacement, and continuous positive airway pressure. Indications for stent placement include palliation, as a bridge to surgery, in patients with high surgical risk, or coverage of post-surgical anastomotic dehiscence. Tracheostomy is a last resort.

#### Tracheal diverticulosis

**Fig. 10:**
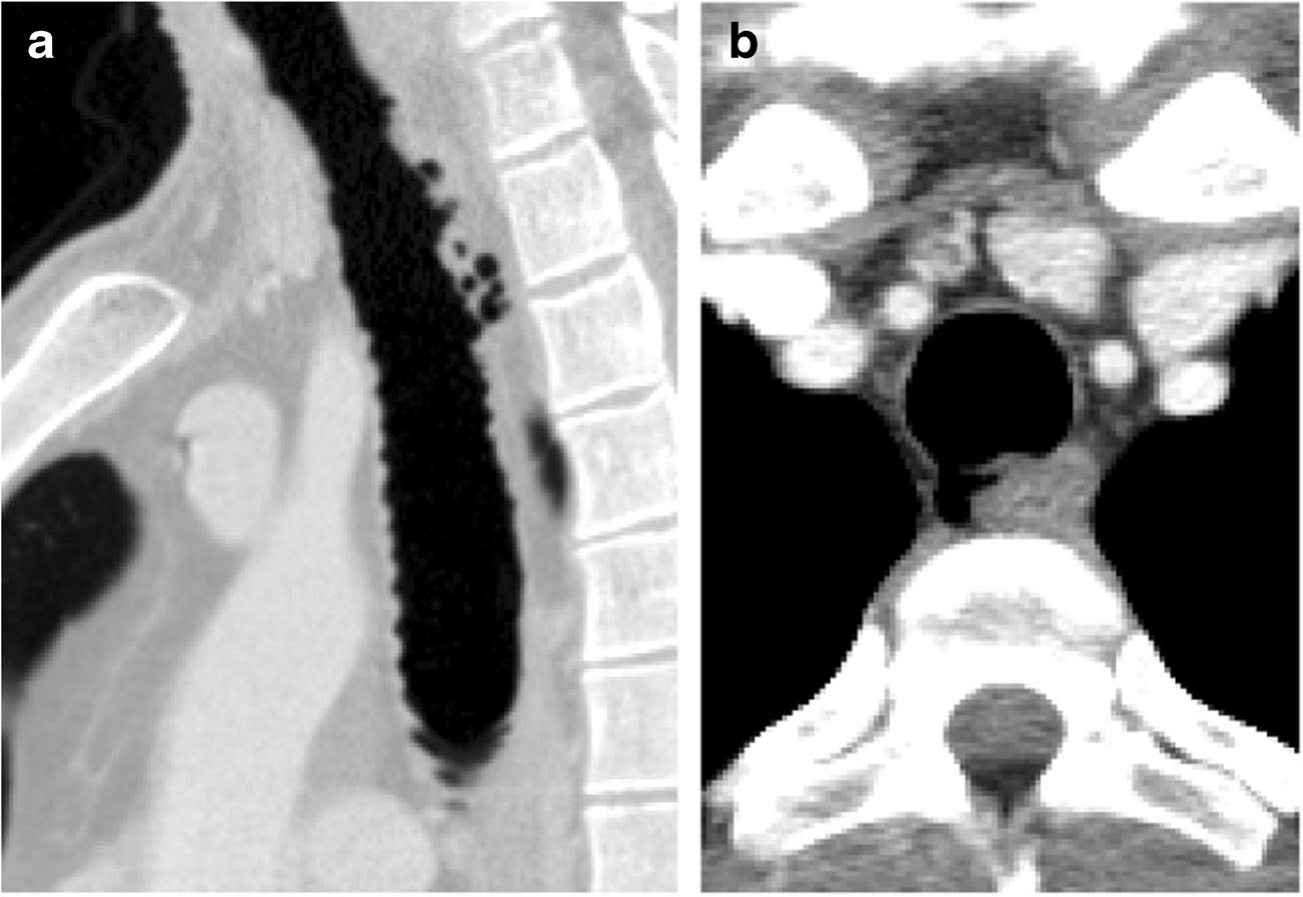
Thirty-nine-year-old man with **a** sagittal and **b** axial CT imaging demonstrating innumerable small diverticuli and corrugated appearance of the trachea compatible with tracheal diverticulosis

Tracheal diverticula can be congenital or acquired. The congenital tracheal diverticulum is typically smaller and possesses all layers of tracheal soft tissue, whereas the acquired type is larger, wider, and has a wall consisting solely of respiratory epithelium. An acquired tracheal diverticulum can be an associated finding in patients with chronic cough, such as occurs with chronic obstructive pulmonary disease. This entity typically occurs at a weak point in the tracheal wall, most often along the posterolateral trachea at the junction of the cartilage and membranous portions [17]. It is typically only a few millimetres in diameter (Fig. 10).

### Benign – diffuse – narrowing – with wall thickening – acute

#### Infection

**Fig. 11:**
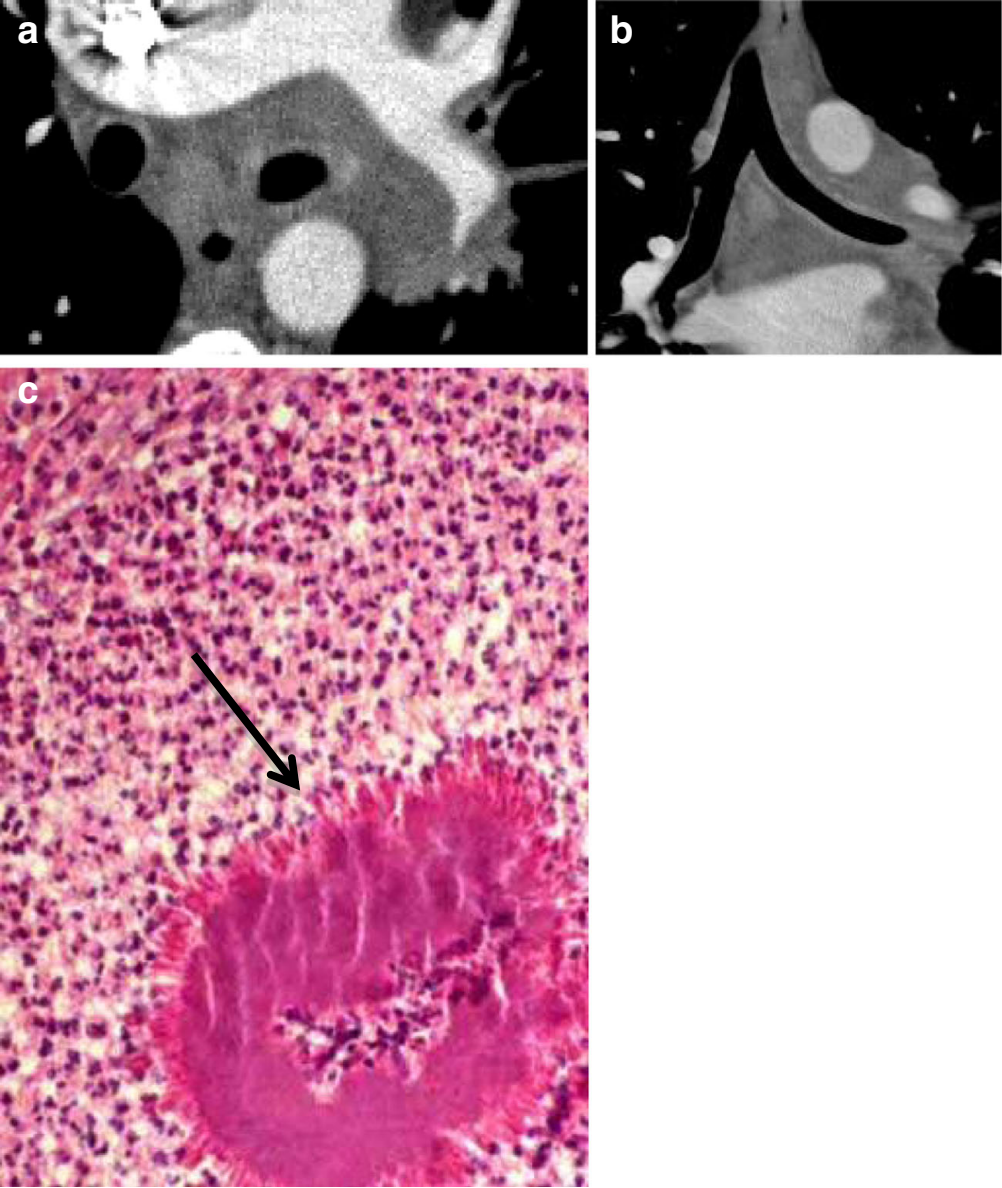
Thirty-four-year-old man with **a** axial and **b** coronal contrast enhanced CT imaging demonstrating matted mediastinal lymphadenopathy surrounding the left lower lobe bronchus with mild extrinsic narrowing and an irregular lesion of the left main bronchus. **c** Pathology reveals acute inflammatory exudate and *Actinomyces* colony, note characteristic sulfa granule (*arrow*)

There are several infections that involve the tracheobronchial tree. Endobronchial tuberculosis is found in 10–40 % of patients with active infection. Approximately 90 % of patients with endobronchial tuberculosis have a degree of bronchial stenosis [18]. Another example of endobronchial infection is actinomycosis. Actinomycosis is an anaerobic gram positive bacteria commonly found in the normal oropharynx. Airway infection is most likely a result of aspiration of oral bacteria. If not treated, infection may spread beyond usual anatomic barriers. Endobronchial actinomycosis may be associated with broncholithiasis in the case of secondarily infected broncholiths [14]. On imaging, there may be mild extrinsic narrowing and associated reactive mediastinal lymphadenopathy (Fig. 11).

#### Inflammation (Bronchitis/Bronchiolitis)

**Fig. 12:**
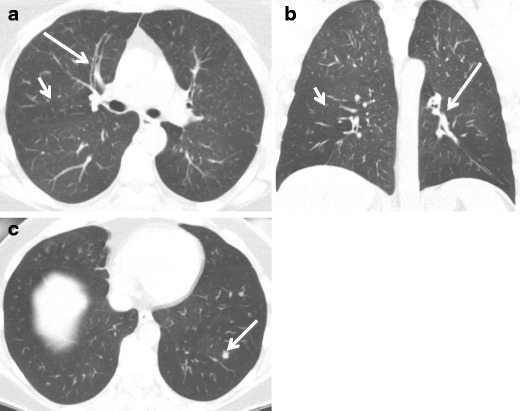
Fifty-one-year-old woman with **a** axial and **b** coronal CT images demonstrating bronchial wall thickening and obstruction (*long arrows*) and areas of mosaic attenuation consistent with regions of air trapping (*short arrows*). There are also **c** groundglass nodules (*arrow*) consistent with bronchiolitis

Bronchitis (inflammation of the large airways) and bronchiolitis (inflammation of the small airways) may be inflammatory or fibrotic (Fig. 12). The inflammatory form may be secondary to many causes, including infection, hypersensitivity, or interstitial lung disease [19].

### Benign – diffuse – narrowing – with wall thickening – chronic

#### Granulomatosis with polyangiitis (Wegener granulomatosis)

**Fig. 13:**
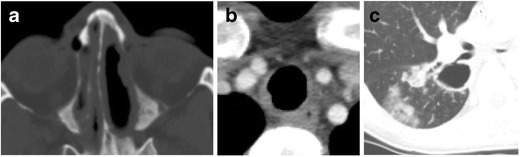
Twenty-nine-year-old woman with history of granulomatosis with polyangiitis. Axial CT images demonstrate **a** nodular thickening of the mucosa within the paranasal sinus, **b** eccentric soft tissue thickening of the tracheal wall, and **c** cavitary pulmonary nodules

Granulomatosis with polyangiitis is a systemic necrotizing granulomatous vasculitis commonly affecting the upper and lower respiratory tracts as well as the kidneys [3, 5, 6, 11, 12, 14, 15, 20]. The trachea and/or bronchi are involved in approximately 50 % of these patients and involvement of the subglottic region is common [15]. The diseased portions of the trachea are seen on CT as circumferential mucosal thickening, ulceration, and irregular nodules (Fig. 13). Additional airway and pulmonary findings including parenchymal nodules, cavitary nodules, and pleural effusions can aid in the diagnosis.

#### Amyloidosis

**Fig. 14:**
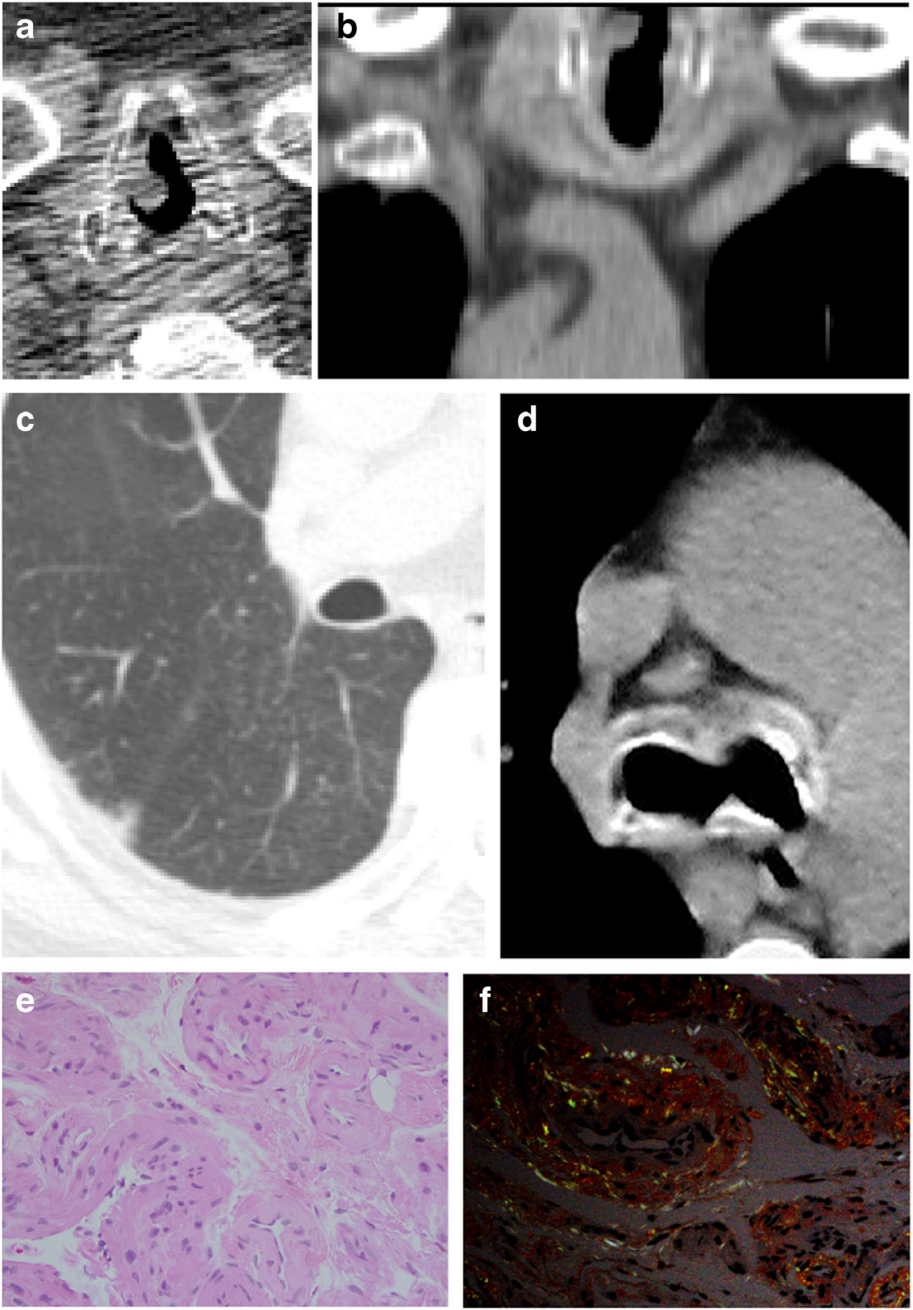
Seventy-three-year-old man with history of amyloidosis. CT imaging in soft tissue and lung windowing demonstrates **a** axial and **b** (finding at the uppermost slices of the chest CT) coronal a single polypoid nodule at the level of the vocal cord, **c** multiple small subcentimeter nodules in a miliary pattern within the lung, and **d** wall thickening and calcification of the proximal bronchi. **e** H&E and **f** Congo red pathology slides reveal amyloid deposits with apple green birefringence under polarized light

Amyloid is an insoluble fibrillary protein which may form extracellular deposits in multiple locations [15]. Pulmonary amyloidosis can be found in three forms including diffuse interstitial deposits, pulmonary nodules, and most frequently, submucosal tracheobronchial deposits [6, 14]. In the tracheobronchial form, these deposits are often found circumferentially and do not spare the posterior wall, unlike tracheobronchopathia osteochondroplastica [3, 12, 13]. Mural calcifications are common [11]. CT appearance may include irregular wall thickening and nodularity [14] (Fig. 14).

#### Sarcoidosis

**Fig. 15:**
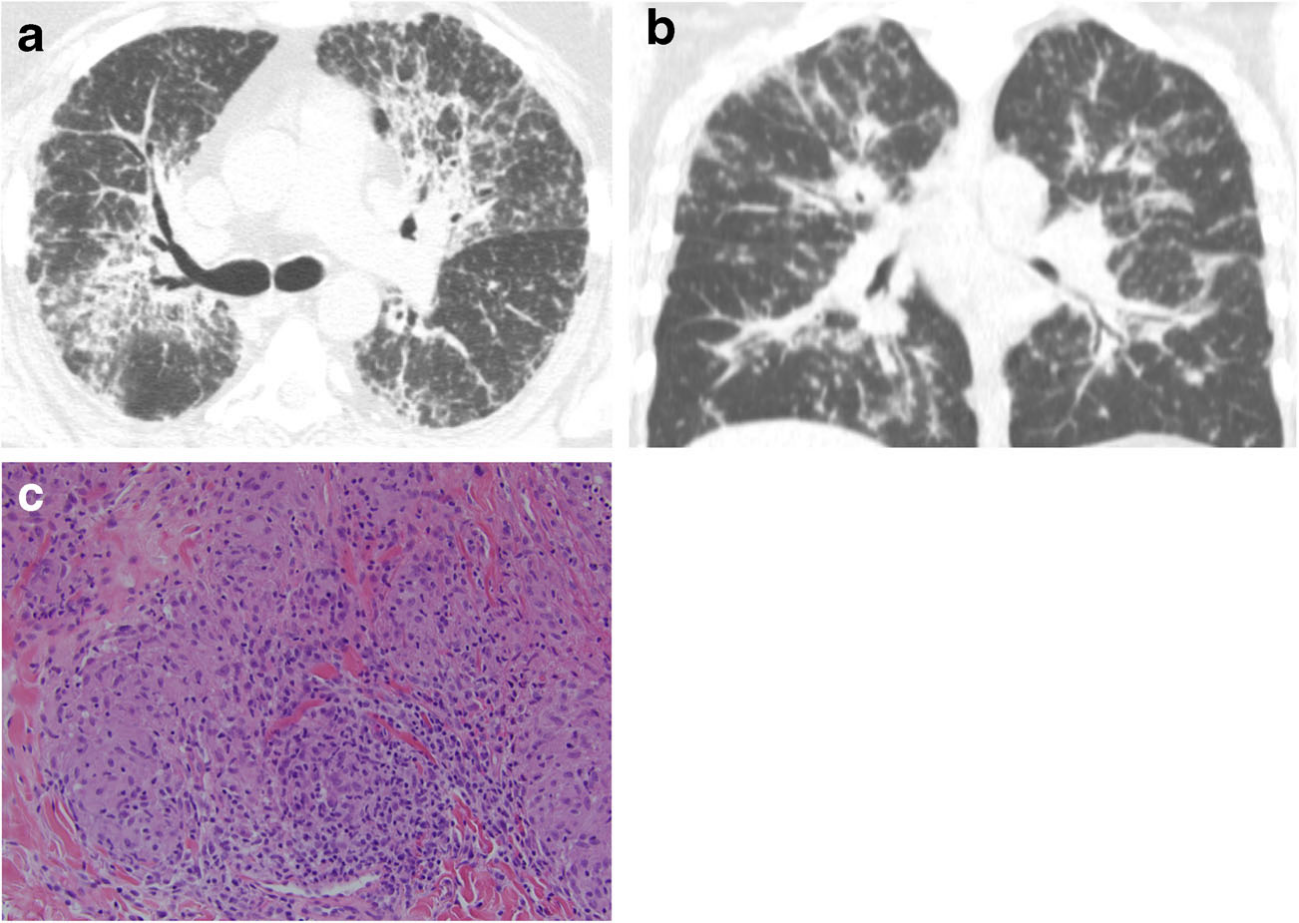
Forty-one-year-old woman with sarcoidosis and **a** axial and **b** coronal CT demonstrating peribronchial thickening and nodularity. **c** Pathology reveals non-necrotizing granuloma with scattered lymphocytes at the periphery

Sarcoidosis is a systemic disease that usually manifests as multiple non-caseating granulomas of the mediastinal and hilar lymph nodes and lungs [3], and rarely in the trachea [5, 14] (Fig. 15). Airway compromise may result from extrinsic compression by lymphadenopathy or intrinsic infiltration of the tracheobronchial walls, with involvement of the bronchi more commonly seen than the trachea [3, 6, 11, 12, 14].

#### Tracheobronchopathia osteochondroplastica

**Fig. 16:**
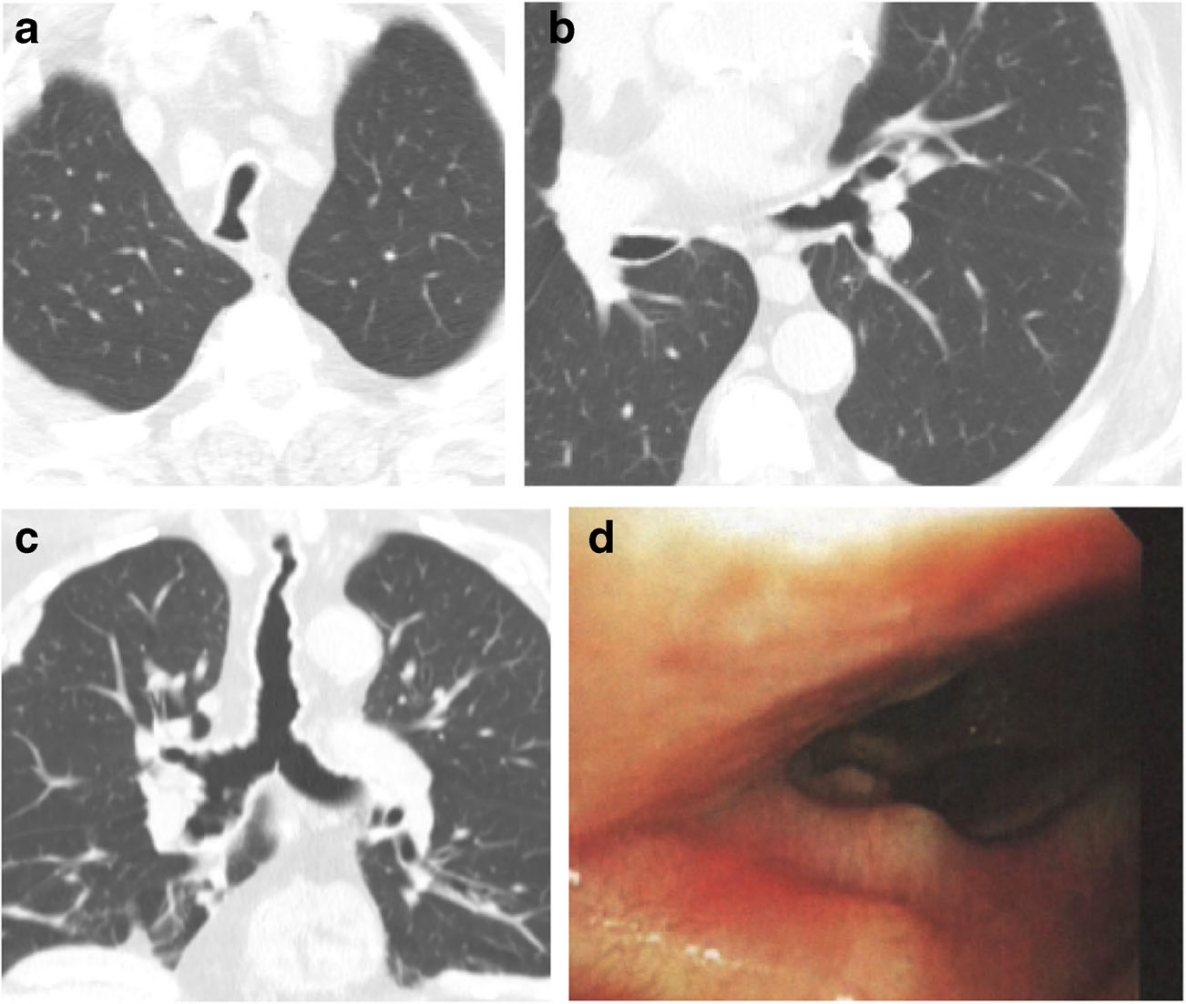
Eighty-two-year-old man with axial **a**, **b** and coronal **c** CT imaging demonstrating narrowed tracheal caliber and irregularity of the walls of the trachea, extending into the bilateral mainstem bronchus compatible with tracheobronchopathia osteochondroplastica. **d** Video still at the time of bronchoscopy demonstrating luminal narrowing of the trachea

Tracheobronchopathia osteochondroplastica is characterized by submucosal osseous and cartilagenous deposits arising from tracheobronichial enchondromas anterolaterally, sparing the posterior membrane [3, 11–13], unlike amyloidosis (Fig. 16). It classically affects the lower two thirds of the trachea [3]. Tracheobronchopathia osteochondroplastica has a slight predilection for men and typically occurs in the sixth decade [11, 14].

### Benign – diffuse – widening

#### Tracheobronchomegaly (Mounier-Kuhn)

**Fig. 17:**
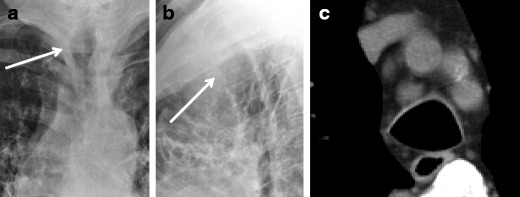
Fifty-nine-year-old man with **a** frontal, **b** lateral radiographs, and **c** axial CT demonstrating a dilated trachea (*arrows*) measuring greater than 3 cm in diameter. These findings are compatible with tracheobronchomegaly

Tracheobronchomegaly (including Mounier-Kuhn syndrome) consists of dilation of the trachea greater than 3 cm and the mainstem bronchi greater than 2.4 cm [3] (Fig. 17). It can be caused by atrophy of the longitudinal fibres and decreased thickness of the mucosa. The congenital form (Mounier-Kuhn syndrome) is caused by congenital deficiency of smooth muscle and elastic fibers [14], and can be seen with Ehlers-Danlos and cystic fibrosis [13]. Expiratory studies may reveal airway collapse [12, 13]. There is an association with tracheal diverticula, which may result in a corrugated appearance on CT [12].

#### Bronchiectasis

**Fig. 18:**
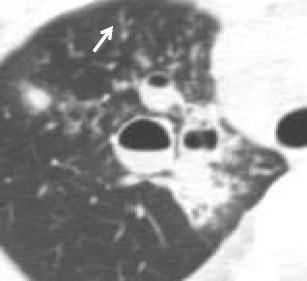
Twenty-one-year-old man with axial CT image demonstrating saccular bronchiectasis with tree-in-bud opacity (*arrow*), debris, and mucous plugging

Bronchiectasis represents a dilation of the bronchi usually secondary to infection, obstruction, or traction. It is often associated with allergic bronchopulmonary aspergillosis, in which case it will frequently be located in the proximal upper lobes. However, the lower zones are the most common location for bronchiectasis. Imaging demonstrates the classic “signet ring” appearance of bronchi larger than adjacent vessels [21] (Fig. 18). Types of bronchiectasis include saccular/cystic, cylindrical, and varicose [22].

### Malignant – focal – narrowing – primary

The symptoms of a tracheal neoplasm are nonspecific and include cough, wheeze, hemoptysis, and stridor. Dysphagia and recurrent laryngeal nerve paralysis can indicate involvement of adjacent structures. Primary malignancies of the trachea are rare and account for only 0.1 to 0.4 % of all malignancies in adults. However, 80–90 % of tracheal tumours are malignant [23]. Primary malignant tumours can be classified by tissue subtype such as surface epithelium, salivary gland, and mesenchymal tissues. The most common histological types are squamous cell carcinoma and adenoid cystic carcinoma.

#### Squamous cell

**Fig. 19:**
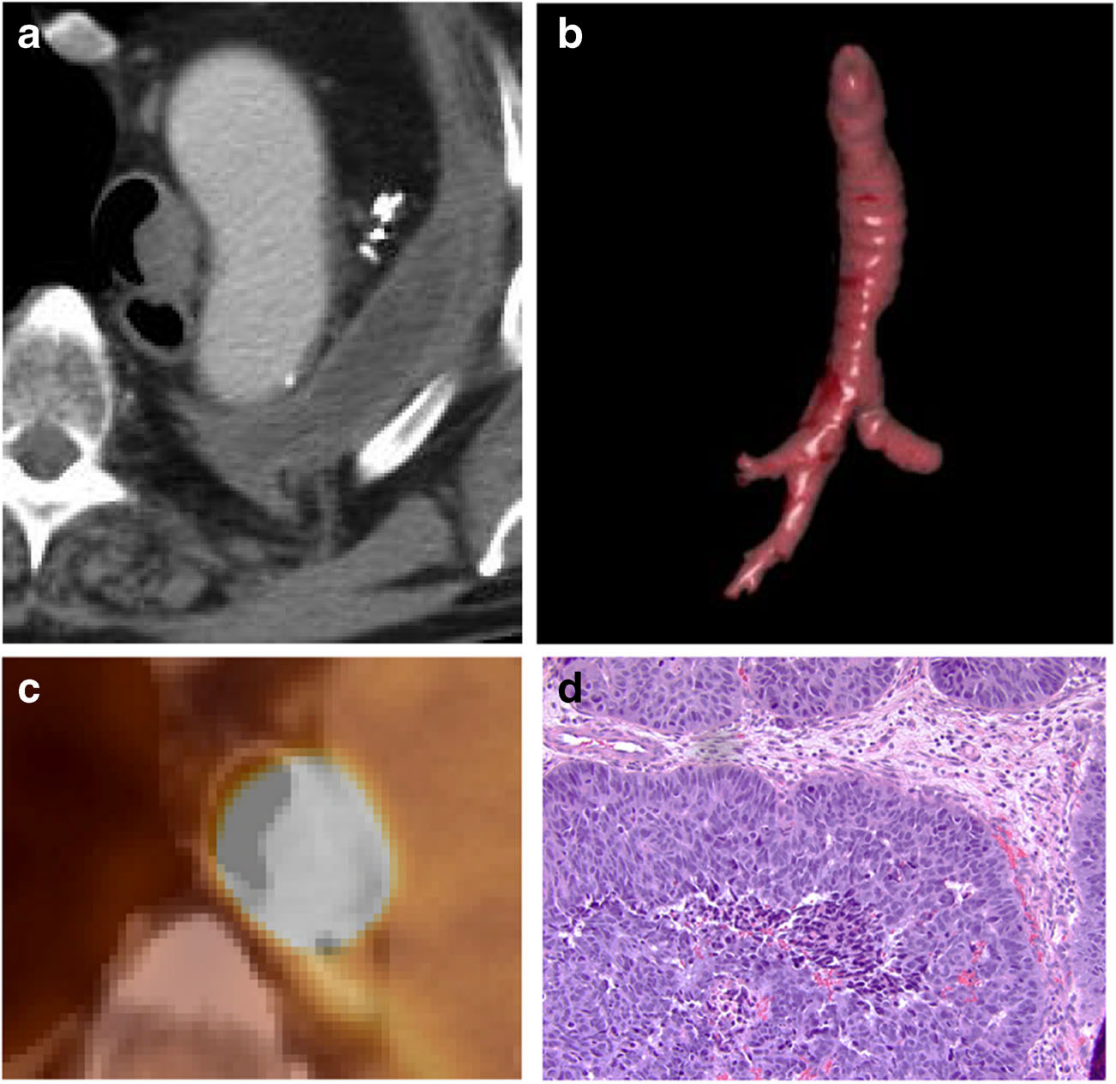
Sixty-six-year-old man with past medical history of squamous cell lung carcinoma. **a** Axial CT demonstrates left posterolateral tracheal wall mass. **b** 3D VR image demonstrates focal narrowing of the distal trachea. **c** PET/CT demonstrates high FDG avidity. **d** Pathology reveals invasive squamous cell carcinoma with basaloid morphology

Squamous cell carcinoma has a strong association with smoking and occurs more commonly in men in their sixth to seventh decades [23]. While it can occur at any location in the trachea, it often occurs in posterior wall of the lower one third of the trachea. One third of patients will have either pulmonary or mediastinal metastatic disease at the time of diagnosis. Additionally, there is an association with carcinoma of the oropharynx, larynx, and lung [24]. Squamous cell carcinoma can have variable appearance on CT imaging, appearing polypoid or sessile, eccentrically narrowed or with mucosal thickening (Fig. 19). It demonstrates high FDG uptake on PET imaging.

#### Adenoid cystic carcinoma

**Fig. 20:**
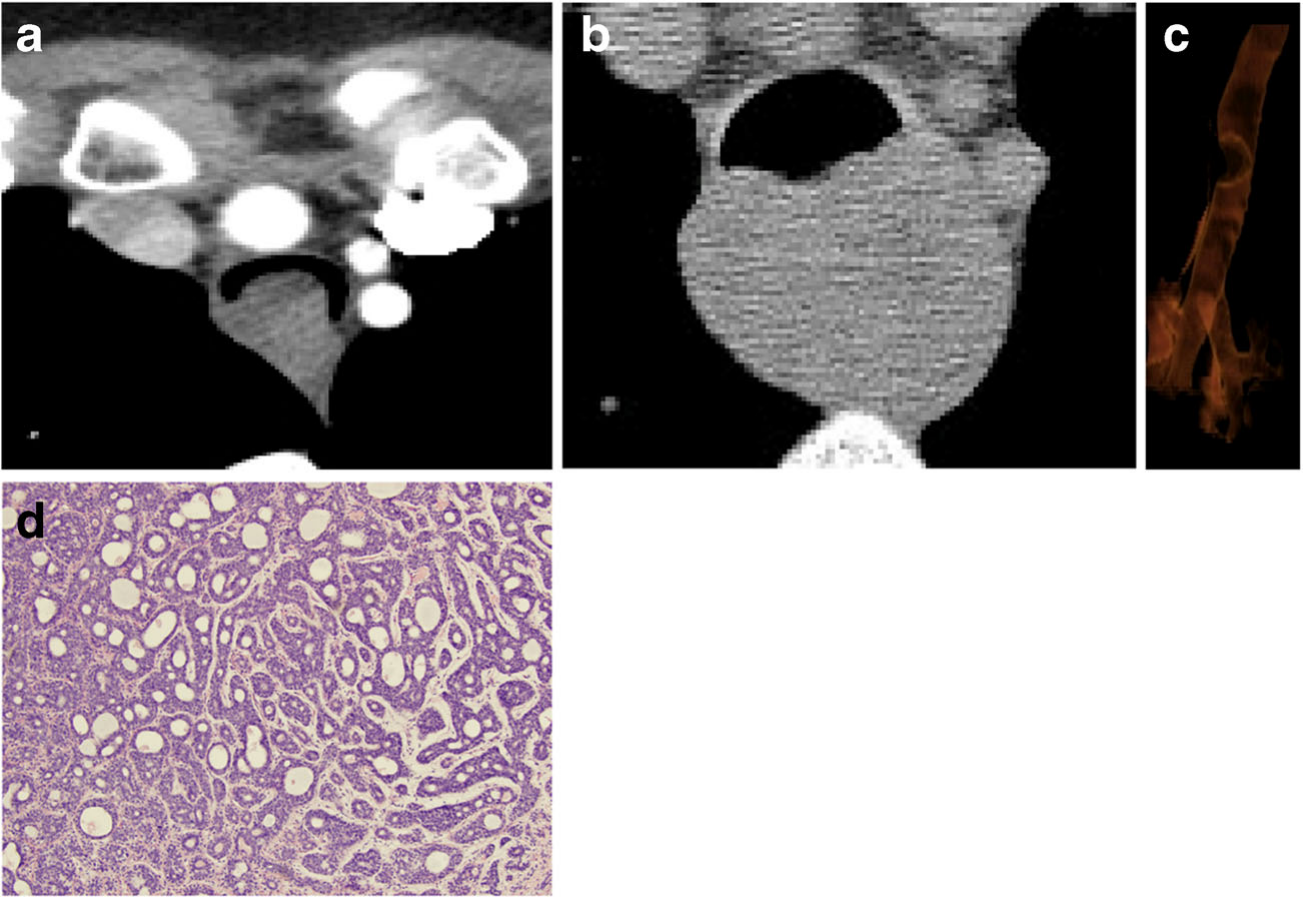
Fifty-year-old woman with a 1-year history of dyspnoea on exertion. Axial **a** contrast and **b** non-contrast CT images demonstrating a large, eccentric soft tissue mass within the trachea. Image **b** demonstrates a large mass with obliteration of the fat plane with the oesophagus. **c** 3D VR image shows significant focal narrowing by the posterior mass with endoluminal extension. **d** Pathology reveals cribriform and microcystic growth patterns of bland basaloid cells with scant cytoplasm and angulated, hyperchromatic nuclei compatible with adenoid cystic carcinoma

Adenoid cystic carcinoma affects men and women equally, occurs most often in the fifth decade, and is not associated with cigarette smoking [25]. It arises from tracheal mucous glands and grows along the submucosa, thus will have intact epithelium with a smooth contour [26] (Fig. 20). Pathologically, these tumours resemble glandular tissue, as they arise from minor salivary glands [27]. It rarely metastasizes and usually is not associated with lymphadenopathy. It can demonstrate high FDG uptake on PET imaging depending on the grade of the tumour. The extent of involvement can be illustrated with multiplanar reconstructions which can help in surgical planning.

#### Carcinoid

**Fig. 21:**
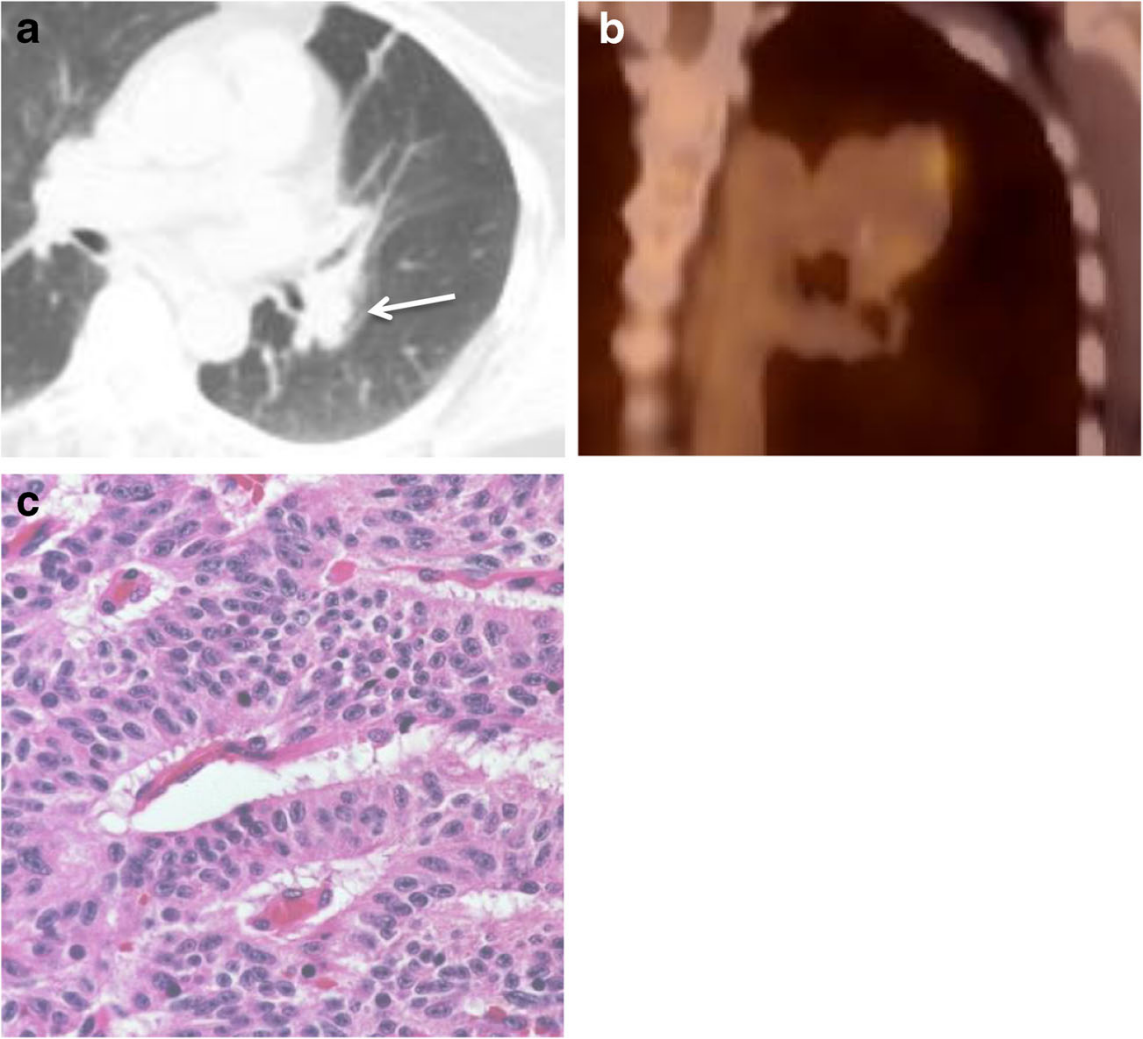
Sixty-nine-year-old woman with **a** axial CT and **b** coronal PET/CT fused images demonstrating a left hilar mass with coarse internal calcifications surrounding the left upper lobe bronchus with intrabronchial extension (*arrow*, figure a). There is heterogenous FDG avidity largely similar to background. **c** Pathology reveals a neuroendocrine tumour with bland uniform cells containing granular (salt and pepper) chromatin compatible with carcinoid

Carcinoid tumors originate from neuroendocrine (Kulchitsky) cells and represent less than 2 % of all tracheobronchial primary malignant neoplasms [26]. Patients typically present in the fifth decade [24] with cough, dyspnoea, and often hemoptysis. Lesions have a usual soft tissue attenuation and frequently have small intraluminal/large extraluminal morphology (Fig. 21). Calcification is seen in a minority of patients. Carcinoid tumours are not associated with a history of smoking. Carcinoid has a high false-negative rate on PET/CT, therefore octreotride scanning is the imaging modality of choice for staging.

#### Primary sarcoma

**Fig. 22:**
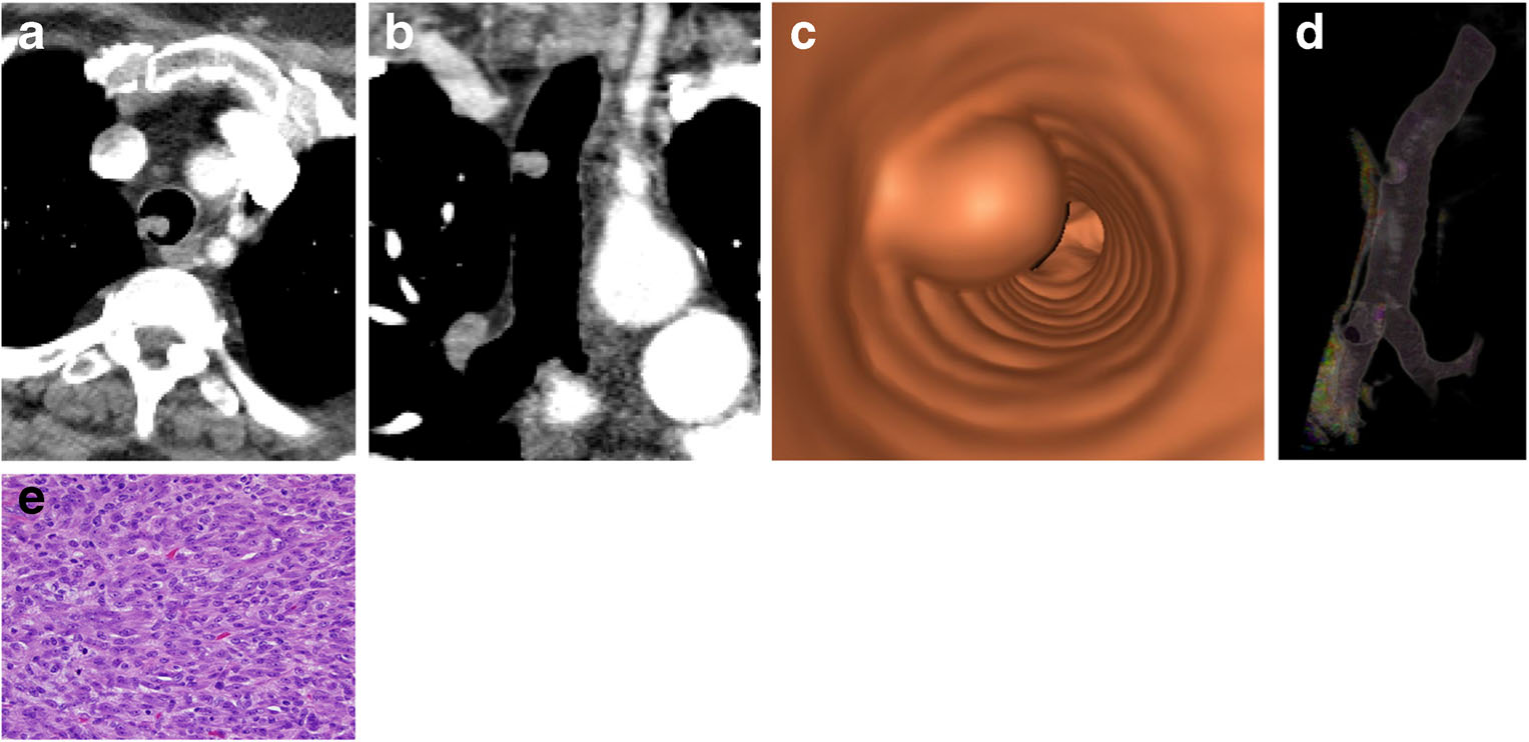
Seventy-two-year-old woman with persistent cough and bloody sputum. **a** Axial and **b** coronal CT demonstrates a polypoid soft tissue mass. **c**, **d** Virtual bronchoscopy and 3D VR image of the mass. **e** Pathology reveals a spindle cell proliferation with mild to moderate nuclear pleomorphism compatible with atypical spindle cell carcinoma

Atypical spindle cell carcinoma is a rare pulmonary neoplasm sometimes referred to as sarcomatoid carcinoma or pleomorphic carcinoma. It is most often seen in middle aged men with a history of smoking and may present on imaging with a polypoid soft tissue mass [28] (Fig. 22). This type of tumour is extremely rare in the trachea.

### Malignant – focal – narrowing – secondary – direct

Metastatic lesions to the trachea can occur from either direct or hematogeneous spread, with direct invasion being more common [24]. The CT imaging is nonspecific as there may be a solitary lesion, multiple lesions, or eccentric wall thickening.

#### Oesophageal

**Fig. 23:**
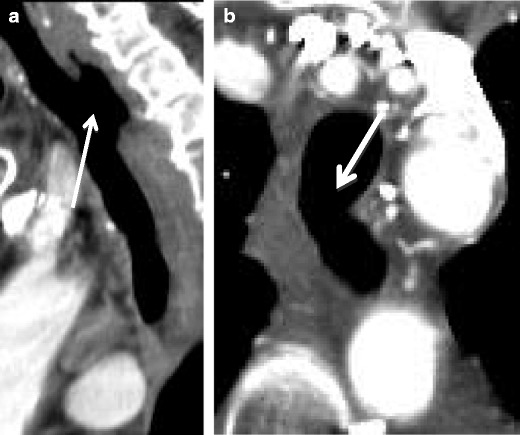
Seventy-three-year-old woman with invasive oesophageal squamous cell carcinoma. **a** Sagittal and **b** axial CTs demonstrate tumour eroding through to the trachea with a wide communication (*arrows*)

Oesophageal carcinoma can lead to the formation of a tracheoesophageal (TE) fistula in 5–10 % of patients (Fig. 23), particularly in those patients who have received radiation therapy. Sixty percent of TE fistulas form as a result of malignancy. Clinical concern for a TE fistula should be raised in a patient with oesophageal carcinoma and recurrent aspiration pneumonia [29].

#### Lung

**Fig. 24:**
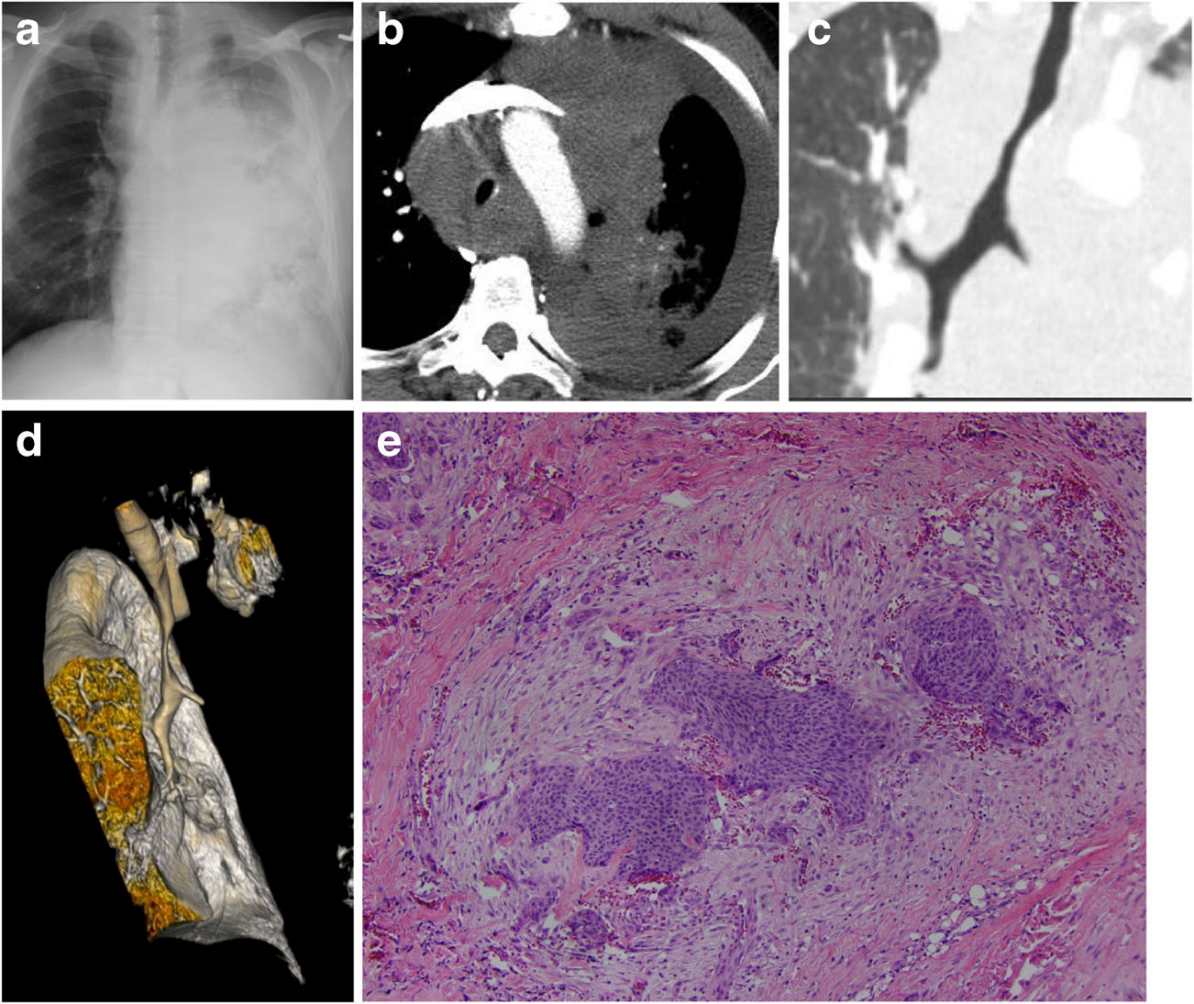
Sixty-one-year-old man with stage IV small cell lung cancer and diffuse involvement of the trachea. **a** Frontal radiograph, demonstrating extensive involvement of the left hemithorax. **b** Axial, **c** coronal, and **d** 3D VR CT images reveal direct invasion and extrinsic compression of the trachea with rightward deviation by the lesion and lymph node mass. **e** Pathology reveals invasive squamous cell carcinoma with desmoplastic stroma

Direct invasion of the trachea by lung carcinoma by definition places the patient in the T4 category of the TNM/American Joint Committee on Cancer (7th edition) lung cancer staging system. Involvement of lymph nodes then determines the staging of T4 lesions as stage IIIA or IIIB. Stage IIIA cancers are a heterogeneous category and some may be resectable, whereas stage IIIB cancers are largely unresectable [30]. CT images may demonstrate direct invasion and extrinsic compression of the trachea and lymph node mass (Fig. 24).

#### Lymphoma

**Fig. 25:**
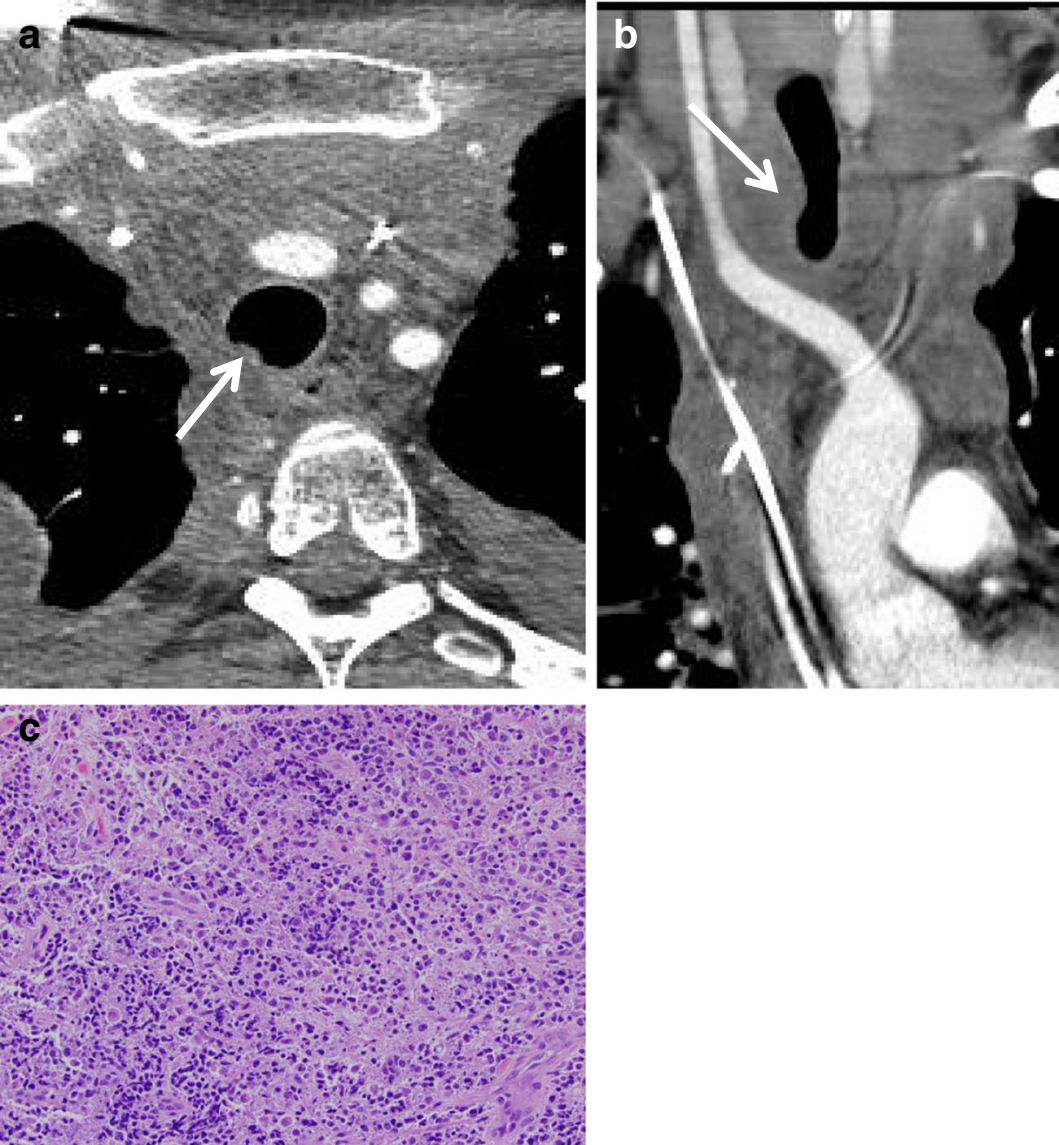
Twenty-five-year-old man with history of large B cell lymphoma of the mediastinum. **a** Axial and **b** coronal CT images demonstrate compression and invasion of the trachea by lymphoma (*arrows*). **c** Pathology reveals malignant cells showing discohesion and apoptosis. Immunohistochemical and flow cytometry results confirm diffuse large B cell lymphoma

Endobronchial metastases of non-Hodgkin lymphoma (NHL) are very rare. Type 1 endobronchial NHL is described as arising from hematogenous or lymphatic routes. Type 2 arises via direct invasion or primarily from bronchus associated lymphoid tissue and is the less common of the two types [31] (Fig. 25).

### Malignant – focal – narrowing – secondary – hematogenous

#### Renal

**Fig. 26:**
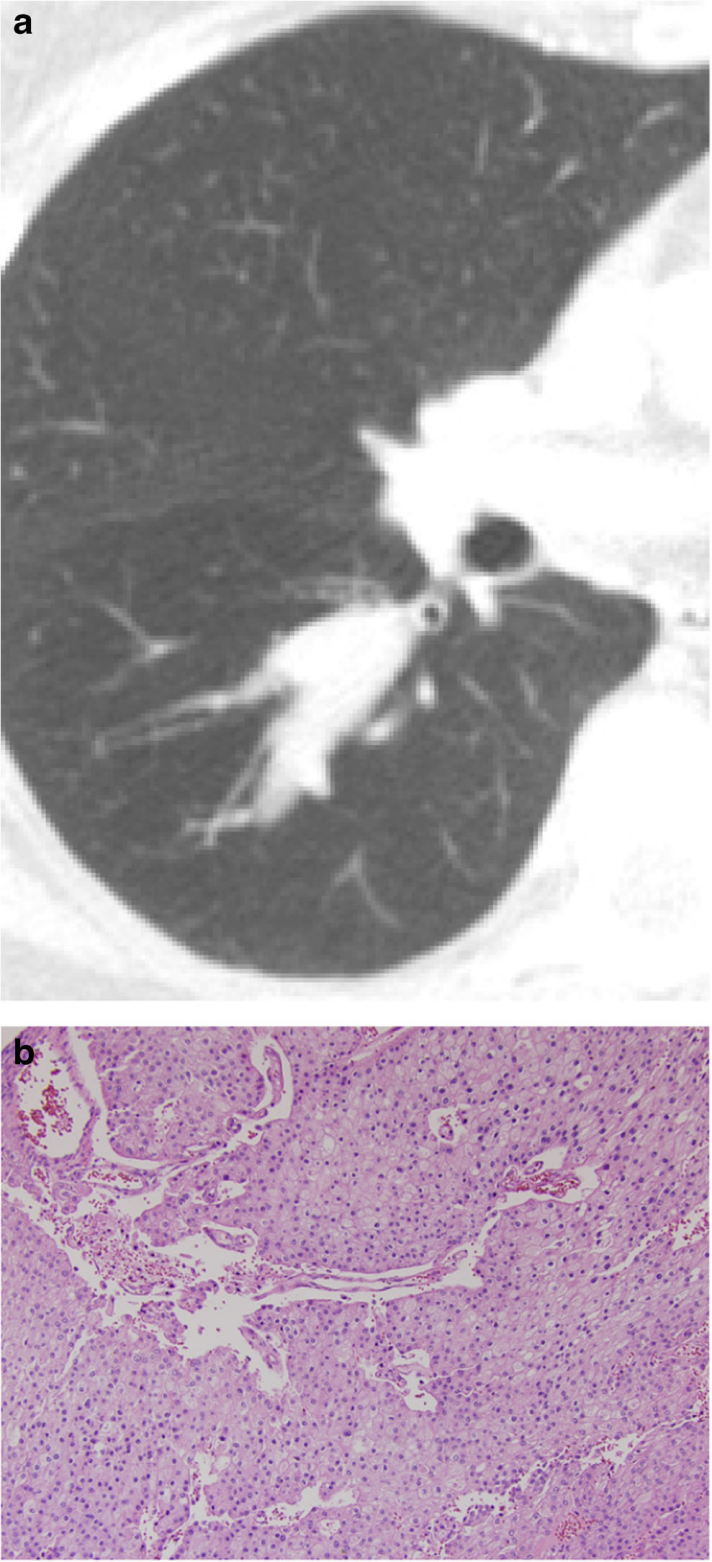
Fifty-five-year-old woman with history of renal cell carcinoma. **a** Axial CT demonstrates right bronchial metastases illustrated by expansion and soft-tissue filling of the airway. **b** Pathology reveals sheets of epithelial cells with eosinophilic cytoplasm, raisinoid nuclei, and perinuclear halo consistent with chromophobe renal cell carcinoma

Renal carcinoma metastasis to the large airways most often manifests with symptoms similar to a primary endobronichial tumour such as hemoptysis [32]. These lesions may appear as a strongly enhancing, high attenuation “finger-in-glove” type mass (Fig. 26).

#### Metastatic melanoma

**Fig. 27:**
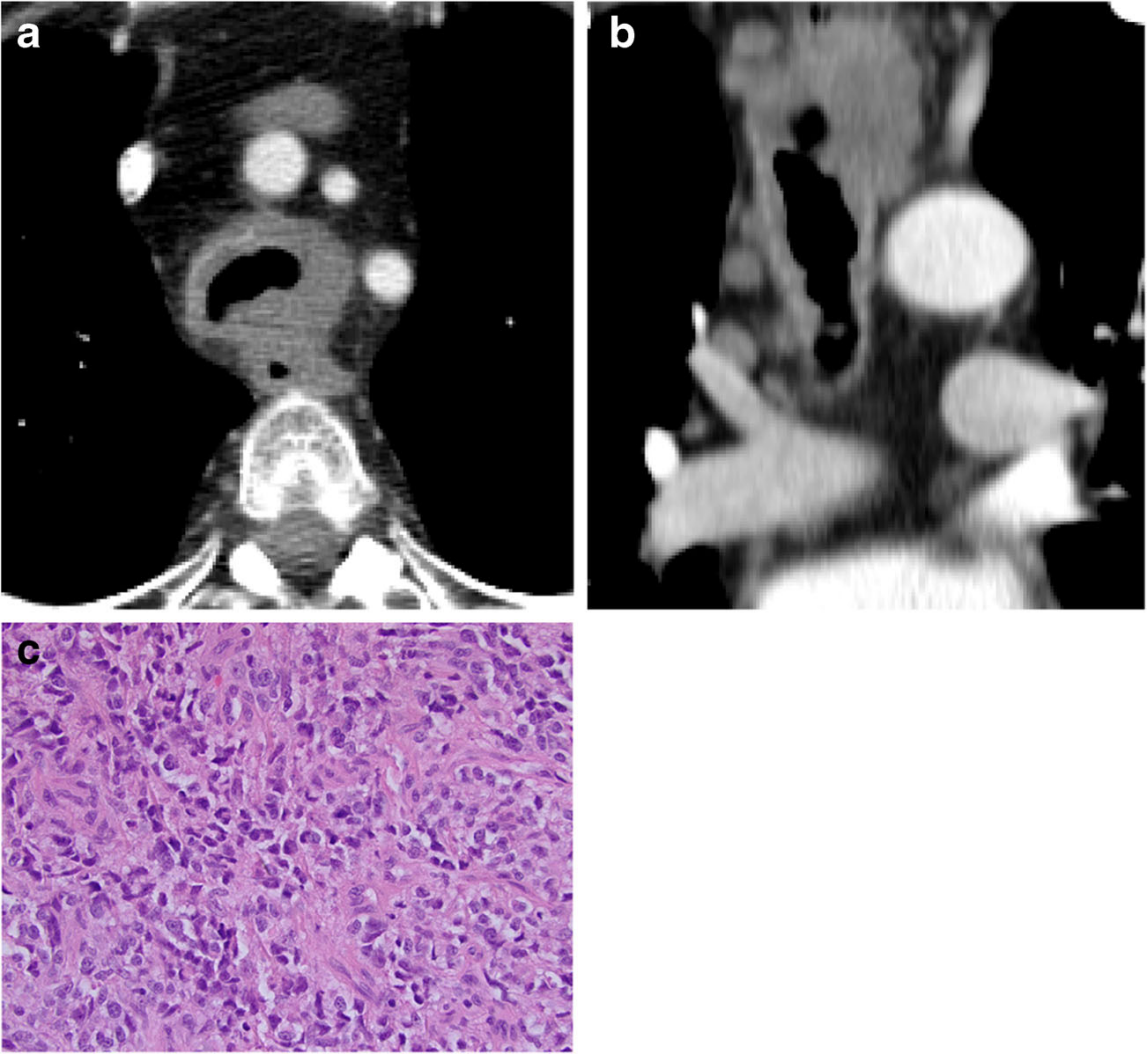
Sixty-eight-year-old man with **a** axial and **b** coronal CT images demonstrating soft tissue thickening, eccentric luminal protrusion, and infiltrative changes of the adjacent mediastinal fat. **c** Pathology reveals malignant cells that are positive for S100, HMB45 and Melan-A compatible with metastatic melanoma

Although the lung is a common site of metastatic melanoma, the bronchi are not. It is a less common source of non-pulmonary endobronchial metastases than breast, colorectal, and renal carcinomas [33]. CT imaging may demonstrate soft tissue thickening and luminal protrusion with associated infiltrative changes (Fig. 27).

#### Kaposi sarcoma

**Fig. 28:**
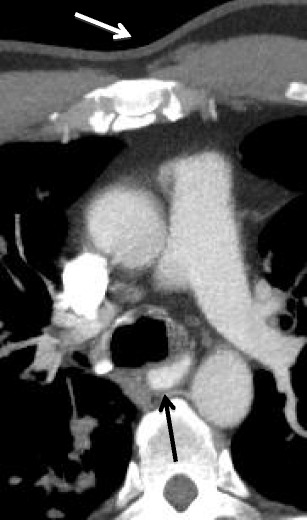
Axial CT demonstrating mucosal thickening with an enhancing lesion of the posterolateral trachea (*black arrow*) as well as overlying chest wall skin thickening (*white arrow*) in a patient with Kaposi sarcoma

Kaposi sarcoma is a multisystemic disorder involving the skin, gastrointestinal tract, lymphatic system, lungs, and tracheobronchial mucous membranes [34]. This case demonstrates diffuse mucosal thickening of the trachea as well as skin thickening (Fig. 28). When mucosal thickening of the trachea is present, it can lead to respiratory compromise which may portend poor overall survival.

## Summary

We have reviewed a wide range of lesions found on imaging of the large airways, lesions that fall into a number of categories, specifically benign versus malignant, focal versus diffuse, and narrowing versus widening of the airway. In addition to this algorithm, evaluation of the circumferential or membranous location of the lesion may also be used to aid in diagnosis, and where appropriate this has been described in the text. Knowledge of the radiologic appearance of these lesions is crucial in developing a differential diagnosis and determining the next step in management. When a pathological specimen is obtained, critical radiologic-pathologic connections can be determined, and diagnostic accuracy may be improved.
